# Musical feedback during exercise machine workout enhances mood

**DOI:** 10.3389/fpsyg.2013.00921

**Published:** 2013-12-10

**Authors:** Thomas H. Fritz, Johanna Halfpaap, Sophia Grahl, Ambika Kirkland, Arno Villringer

**Affiliations:** ^1^Max Planck Institute for Human Cognitive and Brain ScienceLeipzig, Germany; ^2^Institute for Psychoacoustics and Electronic MusicGent, Belgium; ^3^Department of Nuclear Medicine, University of LeipzigLeipzig, Germany

**Keywords:** music therapy, exercise, agency, mood, emotional motor control, jymmin, esthetics

## Abstract

Music making has a number of beneficial effects for motor tasks compared to passive music listening. Given that recent research suggests that high energy musical activities elevate positive affect more strongly than low energy musical activities, we here investigated a recent method that combined music making with systematically increasing physiological arousal by exercise machine workout. We compared mood and anxiety after two exercise conditions on non-cyclical exercise machines, one with passive music listening and the other with musical feedback (where participants could make music with the exercise machines). The results showed that agency during exercise machine workout (an activity we previously labeled jymmin – a cross between jammin and gym) had an enhancing effect on mood compared to workout with passive music listening. Furthermore, the order in which the conditions were presented mediated the effect of musical agency for this subscale when participants first listened passively, the difference in mood between the two conditions was greater, suggesting that a stronger increase in hormone levels (e.g., endorphins) during the active condition may have caused the observed effect. Given an enhanced mood after training with musical feedback compared to passively listening to the same type of music during workout, the results suggest that exercise machine workout with musical feedback (jymmin) makes the act of exercise machine training more desirable.

## INTRODUCTION

A number of factors have been shown to beneficially modulate the performance of motor tasks in combination with music. Acoustic factors in music during a walking task have been shown to modulate the stride length of participants walking in synchrony with the music, thus entraining the speed of beat synchronized walking (Leman et al., 2013). Because the walking was performed in synchrony with the beat and all musical stimuli had the same duration and tempo, the differences in walking speed could only have been the result of music-induced differences in stride length, reflecting the vigor or physical strength of the movement. This is supported by other research showing that participants walked faster on music than on metronome ticks (Styns et al., 2007), and that the sound pressure level of the bass drum in dance music leads to more intense spontaneous hip movements and a higher degree of time entrainment (Van Dyck et al., 2013).

Furthermore, listening to music can have a regulating effect on movement performance in movement disorders such as Parkinson’s disease, where it could be shown that rhythmical auditory cues promote movement speed up to 25% (Thaut et al., 1996; McIntosh et al., 1997) and stride length up to 12% (Thaut et al., 1996). Such a positive effect of musical parameters on the motor behavior of patients with Parkinson’s disease has been reported both immediately during walking (Enzensberger et al., 1997), and as a long-term therapeutical effect (after weeks of training) (Thaut et al., 1996; De Bruin et al., 2010).

Interpersonal proximity in socially shared space will have an automatic effect on the motor behavior of participants and will, for example, increase unintentional coordinated tapping (Wu et al., 2013). Moreover, social interaction in comparison to performing alone or with a simulated partner seems to have a beneficial influence on musical motor performance. When children (of all ages) drum along with either a human partner, a drumming machine, or a drum sound coming from a speaker, they best synchronize in the social condition (Kirschner and Tomasello, 2009), suggesting that a shared representation of a musical goal may modify (in this scenario improve) motor behavior. A modulating influence as observed from social interaction to musical production can also be observed vice versa. Joint music making, including joint singing and dancing, more strongly promoted prosocial behavior such as spontaneous helping and spontaneous cooperative problem solving in 4-year olds (Kirschner and Tomasello, 2010).

Physical workout as a motor activity is known to be healthy, and a therapeutic intervention with physical workout is known to be highly effective for a variety of illnesses and disorders such as depression and anxiety disorders (Ströhle, 2009), multiple sclerosis (Motl et al., 2009), and chronic pain (Vendrig and Lousberg, 1997). It is possible to systematically train distinct muscle groups with specifically developed exercise machines as used in many conventional fitness studios, but also with exercise machines specifically tailored to certain bodily deficits (e.g., after stroke). However, working out with exercise machines is often perceived as rather boring and is rejected by many (Ruby et al., 2011), especially because of the stereotype movements required to do the exercises. Sport performers often listen to music passively to make rather repetitive sports activity more pleasurable. This can have positive effects on sports performance (Terry et al., 2012), which is why athletes often use music in fitness studios and during the preparation of sport competitions (Lim et al., 2009). The musical parameters tempo and rhythm, for example, have been shown to have a motivating and ergogenic influence on performance in sports (Karageorghis et al., 1999; Simpson and Karageorghis, 2006). Furthermore, it has been previously reported that people who listen passively to music while working out may be able use their muscles more effectively (Szmedra and Bacharach, 1998). Supporting the idea that listening to music benefits physical activity, evidence has suggested that multi-year amateur dancing helps preserve cognitive, motor, and perceptual abilities, and thus everyday life competence of elderly individuals (Kattenstroth et al., 2010).

Making music as a typically interactive social activity that involves agency and synchronization has the potential to integrate these factors that are beneficial during motor tasks (as described above). Making music, in comparison to only passively listening to music, thus probably mediates another set of beneficial effects for the performer. It has been reported to have a relaxation effect on performers similar to well-established recreation practices such as progressive muscle relaxation (Kibler and Rider, 1983). It probably has an activating effect on the immune system, as evidenced by measuring Salivary Immunoglobulin A (SlgA) during instrument playing or singing, in comparison to passive music listening (Kuhn, 2002; Kreutz et al., 2004), and an increase of cortisol levels during choir singing. Furthermore, it could be shown that music can increase perceived movement motivation and experienced mood (van der Vlist et al., 2011).

Notably, it has been shown that high energy musical activities seem to more strongly induce a release of endorphins, as measured using pain threshold. Singing, dancing, and drumming all increased the post-activity pain tolerance, and also resulted in elevated positive affect (Dunbar et al., 2012). A recent study explored a novel method to make music while systematically increasing physiological arousal by exercise machine workout (Fritz et al., 2013). This study showed evidence for a massive decrease in perceived exertion when combining workout with musical agency (jymmin) and also an increased motor effectivity when comparing this condition to a control condition where music was listened to passively during workout (similar to conventional training in fitness studios). Note that here we adapt a definition of agency as a performance of bodily movement guided by an agent, and governed by a goal or intention. We employ the term musical agency, because the goal/intention of the musical agency condition is a modulation of musical sounds. In the current study we expected to find effects of musical agency during workout (jymmin) on perceived mood and anxiety compared to passively listening to music during workout. To this end, the same set of machines was used as in the previous study, which allowed for a musical feedback during guided movements on fitness machines.

## MATERIALS AND METHODS

### PARTICIPANTS

Fifty-two participants (27 male) took part in the experiment. The age range was 20–49 years for males (mean = 27.07) and 20–43 years for females (mean = 27.33). Participants used the fitness machines in groups of three. None of the participants were professional athletes, body builders, or musicians.

### EXPERIMENTAL DESIGN

The experiment comprised two conditions: In one condition the participants operated fitness machines while passively listening to music (“passive listening” condition); in a second condition they operated the fitness machines while listening to the musical feedback of their movements (“musical agency” condition). The physical workout was conducted with three different fitness machines, a tower (for a depiction see Fritz et al., 2013), a stomach trainer, and a stepper. All three machines are standard fitness machines that are commercially available from several companies, all allow for guided movements, and feature joints where sensors can easily be attached. The tower features a metal bar attached to weights via a cable. Performers pull down on the metal bar in a well-controlled movement to train the biceps. In the stomach trainer the legs can be fixed in an angled position, and sit-ups are performed, where the movement can be supported by moving the back support of the machine forward while pushing/pulling a semicircular handle with the arms. This way both stomach and arms muscles can be trained, so that the very exhausting sit-up movement can be performed over a longer period of time. The stepper consists of two platforms, one for each foot, so that the performer stands on the machine. Performers push down on the platform with first the left and then the right foot shifting their body weight so that the platforms move accordingly. A resistance is introduced so that the workout becomes more exhausting.

In the “musical agency” condition the movement of the fitness machines was mapped to a musical composition software (Ableton Live 8) so that the deflection of the fitness machines corresponded to musical parameters of an acoustic feedback signal. In the music composition software we prepared a series of musical loops (either wav-audio files or midi sequences), which were set to repeat, and were set to temporally synchronize at a constant tempo of 130 bpm. The style of the music composition used in the experiment was rather simplistic electronic (dance) music. In the composition software, several of the loops could be attributed to one of the “fitness instruments.” For each of these loops, a track with an effect section was created. The movements of each fitness machine were mapped to modulate different parameters for each loop, which were specified in the effects section of each track. Thus, different loops (audio- and midi-) could be influenced by several audio effects by each fitness machine simultaneously. The effects and loops were chosen so that even relatively small movements in the centimeter range created a perceivable musical effect for the performer, culminating in one interesting musical dimension associated with each fitness instrument. Composition effects used in the experiment were bandpass filter (Ableton VST plugin autofilter; the cutoff of the bandpass filter has a strong effect on the perceived timbre of the audio signal; this was used on all three fitness machines), and pitch shift in association with the Ableton VST plugin scale stance filter (allowed for the generation of simple melodies within a scale; this VST plugin was used on the tower). For example, on the tower the cutoff frequencies of two bandpass filters (located in the effects section of two different tracks) were set to modulate a driving techno beat and a bassline (each located in one of the two respective tracks). The cutoff frequencies of the bandpass filters were mapped to the movements of the tower. This meant that in the absence of exerted power, the cutoff was very low (so that no sound except some very deep bass frequencies was audible) and increased in relation to the distance the weights were pulled (so that simultaneously the bassline and the drum loops would blend in their higher frequency spectrum; an effect often used in dance music). While on the bassline this effect is strongly perceived on the low and medium spectral range, on the beat it is also strongly perceived in the high frequency range where cymbals are effected. In the high frequency range additionally the pitch of a midi loop (triggering a synthesizer) was effected, so that a simple melody on a software-synthesizer could be created by moving the weights in the top range of displacement.

We call this musical feedback technology “jymmin,” a cross between jammin and gym. The musical feedback was designed so that the three fitness machines created sounds that could be interactively combined into a holistic musical piece at a constant tempo of 130 bpm. The musical interaction was constrained for its degrees of freedom, by predefining the sounds to be modulated, the parameters to be modulated, and the pulse of the music. Although the musical piece had a clear metric pulse as defined by beat loops manipulated both on the stepper and the tower, the movements of the participants were not strictly coupled to the meter, but could, for example, include slow bandpass filter movements over several (musical) measures.

Note that the musical soundscape in both conditions was comparable, because the music to which participants worked out in the “passive listening” condition was created by musical interactions similar to the “musical agency” condition by nine different groups of participants (ensuring an ecological validity of the passive listening musical baseline signal), which were recorded previous to the experiment. We controlled for the sequence in which the conditions were performed, so that half the participants first performed “passive listening,” and the other half first performed “musical agency.”

### EXPERIMENTAL PROCEDURE

The participants from all groups met for the first time during the experiment and were asked to choose their preferred fitness machines. The task for all conditions was identical: “Use the fitness machines in a way in which you are physically comfortable.” All participants filled out general information questionnaires to assess sex and age, the short form of the Multidimensional Mood Questionnaire (MDMQ; Mehrdimensionaler Befindlichkeitsfragebogen; Steyer et al., 1997), and the State-Trait-Anxiety-Inventory (STAI; State-Trait-Angstinventar; Laux et al., 1981) to assess a baseline of their mental state before the experiment. Each condition was performed for 10 min; a speaker system was used so that the sound was heard by all participants. Although 10 min of fitness training may seem a rather short duration (often fitness training is undertaken for 15–20 min), we chose this shorter time frame because (1) participants perform two conditions, so the time adds up to 2 × 10 = 20 min and we did not want to over-exert participants, and (2) we wanted to avoid the risk that a too lengthy musical piece might with time become boring for the participants (an extra long disco mix track usually lasts a maximum of around 10 min). Each condition was followed by a 10 min break to relax and again fill out the STAI questionnaire; additionally after each condition the Multidimensional Mood Questionnaire (MDMQ; Mehrdimensionaler Befindlichkeitsfragebogen; Steyer et al., 1997) was filled out. The MDMQ is a mental state assessment instrument that measures the current mental state with three bipolar designed subscales (good/bad mood, alertness/fatigue, calmness/restlessness) with a total of 12 items. Each subscale consists of four adjectives, of which two belong to the negative (e.g., “bad,” “uncomfortable”) and two to the positive (e.g., “well,” “satisfied”) pole. These items are rated on a five-point Likert-scale with one (“not at all”) to five (“very”). The MDMQ is designed for adolescents as well as adults and can be used to evaluate the progress of mood influencing therapies and interventions. The short form of the test, which is used here can be filled out in less than 6 min and the possible scores are between 12 (bad mental state) and 40 (good mental condition). Despite the brevity of the MDMQ, it has been shown to be highly reliable (Heinrichs and Nater, 2002), and the internal consistency (Cronbach’s alpha) of the short form scales are between α = 0.73 and α = 0.89. The STAI can measure both anxiety as a trait and anxiety as a state. For the current study we were interested in measuring state anxiety. The questionnaire consists of 20 items describing the current mental state of the participants (for example “I feel nervous.” or “I feel relaxed.”). The items are rated on a four-point-Likert scale from one (“not at all”) to four (“very”). The questionnaire is well approved in research and clinical practice and shows high internal consistency (Cronbach’s alpha of α = 0.90).

### DATA ANALYSIS

The behavioral data were analyzed using SPSS 18 (IBM). Participants who had missing responses because a questionnaire was filled out incompletely were excluded from the analysis. In total, data from the MDMQ were analyzed for 45 participants and data from the STAI were analyzed for 51 participants.

In order to obtain mean scores for each subscale on the MDMQ, responses to the four questions corresponding to each subscale were averaged. These mean scores range from 1 to 5, reflecting the one to five Likert scale used to rate each item. Items belonging to the negative pole (e.g., “bad” or “uncomfortable”) were reverse scored, (e.g., higher scores on items relating to bad mood result in a lower score). Therefore, a higher mean score on the “good vs. bad mood” subscale corresponds to a better mood, a higher mean score on the “calmness vs. agitation” subscale indicates a greater degree of calmness, and a higher mean score on the “alertness vs. tiredness” subscale indicates a higher level of alertness.

A multivariate analysis of variance (MANOVA) with repeated measures was performed to test the effect of condition (musical agency vs. passive listening) and condition order (passive listening first vs. musical agency first) on the linear combination of the three subscales of the mood and activation questionnaire which was rated after each condition. Condition was a within-subjects variable and condition order varied between subjects. Follow-up univariate analysis of variance (ANOVA) was performed for each of the three subscales to determine whether differences in mood or differences in activation were driving the effect.

A repeated-measures ANOVA was also carried out to test the effect of condition (baseline, musical agency, and passive listening) and condition order (passive listening first vs. musical agency first) on anxiety, as measured by the STAI.

## RESULTS

A MANOVA revealed that the main effect of condition on the three mood subscales was statistically significant, Pillai’s Trace = 0.21, *F*(3, 41) = 3.56, *p* < 0.05. The main effect of condition order was not significant, Pillai’s Trace = 0.91, *F*(3, 41) = 1.36, *p* = 0.27; the interaction between condition and condition order, however, was significant, Pillai’s Trace = 0.23, *F *(3, 41) = 4.10, *p* < 0.05.

**FIGURE 1:**
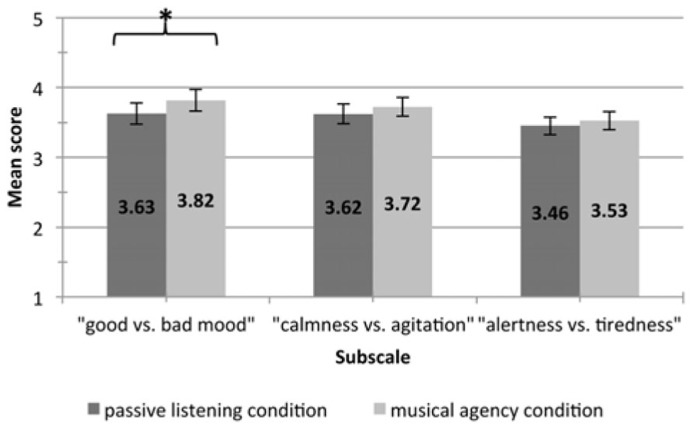
The figure depicts mean ratings for each Multidimensional Mood Questionnaire subscale by condition; error bars show the standard error and *indicates a significant univariate effect with a *p*-value of <0.05.

Follow-up univariate ANOVAs showed a significant main effect of condition on the “good vs. bad mood” subscale, *F*(1, 43) = 10.67, *p* < 0.05. As shown in **Figure 1**, the mean score on this subscale was higher following the “musical agency” condition (*M* = 3.63, SD = 0.99) than following the “passive listening” condition (*M* = 3.82, SD = 1.03). However, condition did not have a significant main effect on either the “calmness vs. agitation” subscale, *F*(1, 43) = 1.83, *p* = 0.18 or the “alertness vs. tiredness” subscale, *F*(1, 43) = 0.46, *p* = 0.50.

**FIGURE 2:**
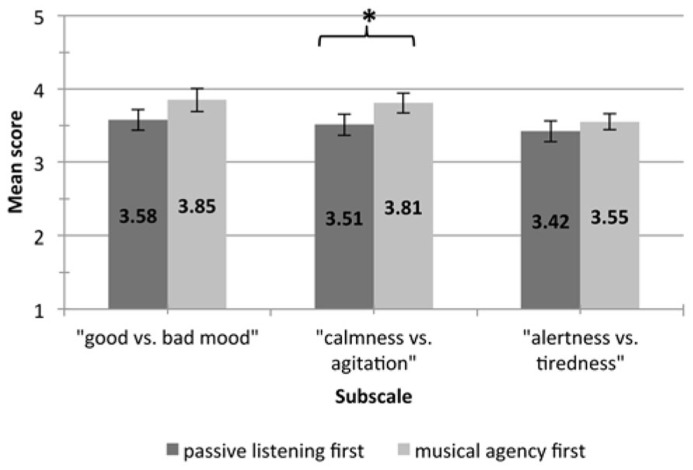
The figure depicts mean ratings for each Multidimensional Mood Questionnaire subscale by condition order; error bars show the standard error and *indicates a significant univariate effect with a *p*-value of <0.05.

The univariate main effect of condition order was significant for the “calmness vs. agitation” subscale, *F*(1, 43) = 4.12, *p* < 0.05. As **Figure 2** shows, participants scored higher on this subscale when they performed the “musical agency” condition first (*M* = 3.81, SD = 0.94) than when they performed “passive listening” first (*M* = 3.51, SD = 0.95). There was no significant effect of condition order for the “good vs. bad mood” subscale, *F*(1, 43) = 2.34, *p* = 0.13 or for the “alertness vs. tiredness” subscale, *F*(1, 43) = 2.50, *p* = 0.12.

**FIGURE 3:**
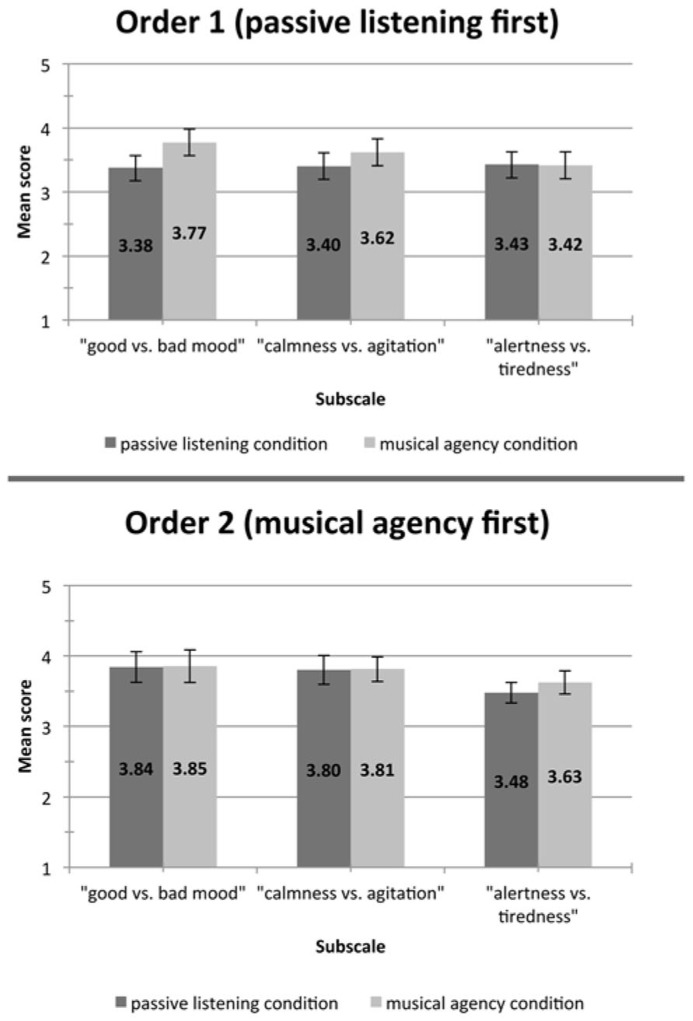
**The figure depicts mean ratings for the “good vs. bad mood” subscale by condition and condition order; error bars show the standard error**.

The univariate interaction between condition and condition order was significant for the “good vs. bad mood” subscale, *F*(1, 43) = 8.24, *p* < 0.05. As seen in **Figure 3**, the difference between the “passive listening” condition and the “musical agency” condition was greater when the “passive listening” condition came first. There was no significant interaction for the “calmness vs. agitation” subscale, *F*(1, 43) = 1.10, *p* = 0.30, or for the “alertness vs. tiredness” subscale, *F*(1, 43) = 1.03, *p* = 0.32.

**FIGURE 4:**
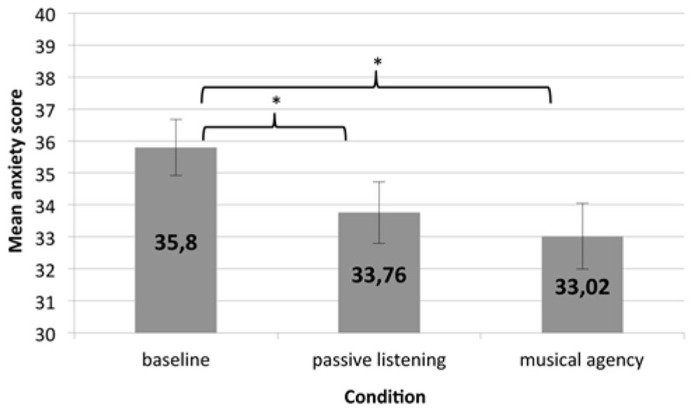
The figure depicts mean anxiety scores on the State Trait Anxiety Inventory by condition; error bars show the standard error and *indicates a significant effect with a *p*-value of <0.05.

Results for the anxiety questionnaire ANOVA showed a significant effect of condition, *F*(2, 98) = 8.32, *p* < 0.05. The mean STAI scores for each condition can be seen in **Figure 4**. Tests of within-subject contrasts revealed that both “passive listening” and “musical agency” lowered anxiety relative to the baseline. Anxiety scores after “passive listening” (*M* = 33.76, SD = 6.88) were significantly lower than the baseline (*M* = 35.8, SD = 6.28),* F*(1, 49) = 7.54, *p* < 0.05. Anxiety scores following the “musical agency” condition (*M* = 33.02, SD = 7.33) were also lower than the baseline, *F*(1, 49) = 13.02, *p* < 0.05. However, anxiety scores for the “passive listening” condition were not significantly different from anxiety scores for the “musical agency” condition, *F*(1, 49) = 1.64, *p* = 0.21. The main effect of condition order on anxiety was not significant, *F*(1, 49) = 0.01, *p* = 0.93, nor was the interaction between condition and condition order, *F*(2, 98) = 1.04, *p* = 0.357.

## DISCUSSION

The data indicate that musical agency during exercise machine workout can more strongly increase the subjectively perceived mood. Findings from a recent study suggest that the physical exertion during musical performance is proportionate to endorphin release (Dunbar et al., 2012). Authors also reported corresponding changes in positive affect. Note that in the current study we could compare the musical agency condition with a control condition that was comparably high with respect to physiological activity. In fact in a different experiment, which is not reported here where we measured spirometry, we found that physiological activity during a stereotypical workout control condition as used in the current experiment is even higher than during the musical agency condition (Fritz et al., 2013). Thus it appears that the amount of physical exertion (and corresponding endorphin release) does not alone modulate mood in the participants. However, it cannot be excluded that mood is influenced by physiological differences between the conditions related to the different (less stereotype) movement patterns during the active condition (Fritz et al., 2013).

The mechanism by which mood may be enhanced by the musical agency condition is not yet completely apparent, but it is probably related to hormonal processes, which are known to have the capacity to influence mood (Zubieta et al., 2003). The time period between conditions (10 min) is not sufficient for hormones like cortisol and endorphins to return to a baseline level and the observed lingering effect of the musical agency condition (effect of order; **Figure 3**) is likely to be due to such a longer term hormonal effect. The agency condition more strongly creates emotional experience in the performer. In dimensional emotion models physiological arousal is almost always a basic dimension (Fritz, 2009). If such a physiological arousal (systematically created by workout) in the performer is combined with expressional behavior as during the musical agency condition, it may facilitate emotional experience in the performer. This would be further facilitated by the fact that to a great degree musical expression in Western music comprises emotional expression (Fritz et al., 2009). The physiological exercise carried out by the participants did not result in significant differences in the dimensions “calmness vs. agitation” and “alertness vs. tiredness” (two of the subscales of the Multidimensional Mood Questionnaire), but in the “good vs. bad mood” dimension it did. This further underlines the capacity that active music making has an intensifying (ameliorating) emotional effect on the performer.

How may such emotional experience be facilitated during the active condition? Obviously, the musical agency condition is cognitively more demanding than the passive listening condition. It seems to be this additional cognitive challenge in terms of personal expression that brings about the observed mood changes. This observed benefit from higher expressional cognitive demand during the active condition, where participants have control over the music and interact demonstrates that human beings gain from expressing inner thought processes and being creative. This would also indicate why humans are devoted to being social animals. Furthermore it may entail that jymmin may be a convenient method for influencing an asocial mindset.

The finding that anxiety does not differ between the active and passive condition may relate to previous findings showing that making music while modulating positive affect did not influence negative affect (Dunbar et al., 2012) as assessed with the PANAS (Watson et al., 1988). On the other hand, it may be that musical agency does have a capacity to more strongly influence anxiety, but that given the relatively low anxiety of participants to begin with, there is a ceiling effect. It would thus be interesting to repeat the experiment with a high anxiety cohort.

While the exercise movements were guided as a consequence of the design of the fitness machines, they were also less stereotypical than usual during exercise machine workout. This raises the question if it can be regarded a more or less healthy movement than those usually performed on exercise machines. Such are stereotypical movements, which are repeated for a defined number of times (often 10–12) and which are evenly performed from beginning to end, and consist of almost exclusively isotonic movement. In contrast, the current musical feedback technology (jymmin) rather encouraged less stereotypical movement patterns with a greater proportion of isometric movement, for example when participants held the weights at a certain position and someone else tried to play a “solo.”

The idea that movements on exercise machines are most effective when evenly performed from beginning to end and for a defined number of times is largely a historical product, and not proven by physiological data (Hartmann et al., 2010). Given the greater proportion of isometric movements and the smaller stereotypicity, the current movement pattern may be regarded to more closely resemble climbing than exercise machine movement. Moreover, climbing has been argued to be quite healthy (Heitkamp et al., 2005), among other reasons because it is non-stereotypical and accordingly puts less strain on joints than stereotypical movements such as jogging and exercise machine workout.

On a critical note, the musical excerpts listened to in the “passive listening” condition were (similar to those created the in “musical agency” condition) relatively basic musical compositions (electronic music) arranged by non-musicians. Thus it may well be that participants deem other commercially available songs more pleasant to listen to. This is also relevant to the current findings, because it cannot be excluded that the agency in the “musical agency” condition only rendered the listening to the soundscapes less unpleasant than passively listening to them in the “passive listening” condition (and that workout with a neutral soundscape or one’s own favorite music may have more strongly increased the mood than during the musical agency condition). Note however that after the experiment, in a post-experimental interview, participants were asked how they generally felt during the Experiment, and how they liked the music. It was obvious that the large majority of the participants enjoyed the music (also the passive listening part). However, this parameter was unfortunately not quantitatively assessed, so that the above argument has to be regarded a limitation of the current study, and should be considered in future studies. Furthermore, for future experiments it would be helpful to qualitatively assess the experience of the participants in the “musical agency” condition in order to deduce ideas about psychological factors underlying the observed mood effect.

### INDIVIDUAL FACTORS THAT MAY MODULATE THE EFFECT OF JYMMIN ON MOOD

At this point the mechanism by which jymmin has an effect on mood must remain hypothetical. However, there is evidence that during music making, in comparison to passive music listening, a number of body-physiology related parameters are different due to emotion-related autonomic nerve activity (Nakahara et al., 2011), and a greater degree of flow state experience (Wrigley and Emmerson, 2011). It will have to be specified what it is about musical agency that creates the observed psychological effect on mood. We propose that a strong experience of individual expression through music may engage a social communication program that can strongly modulate perception, especially when related to proprioception. Note that the nature and function of such communicative musicality (Malloch and Trevarthen, 2008) has been a focus of recent music research. A manipulation of physiological arousal by music-making-associated workout may enhance the intensity of this experience. The communicative aspect of the musical agency condition is further underscored by the fact that alternating soloing was encouraged by the design of the musical interaction (see above).

Note that it is to be expected that the modulating influence of music making on mood is also influenced by several other factors in the individual that would need to be addressed in future studies: (1) There may be interpersonal factors, such that some performers find each other more (or less) attractive, or friendly. (2) Participants may have in their dynamic interaction a better (or worse) “chemistry” together, which means they may respond more or less sensitively to each other’s actions. (3) The influence on their mood may be modulated by their current mental state for example their daily mood or hormonal disposition (Hunter et al., 2011). (4) The effect on their mood may relate to their openness to experience such a form of rather ecstatic togetherness with others. (5) It may vary depending on how much individuals generally derive enjoyment from musical expression and interaction. (6) It may depend on the musical style of the musical feedback participants produce (if for example they considered it part of their repertoire of favorite music) (Dyrlund and Wininger, 2007; Istók et al., 2013). (7) Finally, the combination of music making and high bodily arousal as conducted in the jymmin music feedback technology also gives rise to the possibility that some individuals derive more positive effect on their mood through physiological experience or disposition. For example, that they can experience greater or lesser reward through a disposition of their dopaminergic system. This would, for example, also relate to patients who suffer from a disorder of the dopaminergic system, as in Parkinson’s disease, or in depression.**

In conclusion, we present a method that combines exercise machine workout and music making, and by this measure makes exercise machine workout more desirable through an influence on experienced mood. A key to its impact on the individual (and thus its potential as a recreational activity and therapeutical approach) may be that movements motivated by social interaction for a common esthetic goal in a different way engage motor control than the stereotypical conventional exercise machine movements. It is discussed which factors in the individual may modulate the effect of this so-called “jymmin.” Furthermore, we outline putative therapeutical benefits of this method.

## Conflict of Interest Statement

The authors declare that the research was conducted in the absence of any commercial or financial relationships that could be construed as a potential conflict of interest.
